# Pyramid Textured Photonic Films with High‐Refractive Index Fillers for Efficient Radiative Cooling

**DOI:** 10.1002/advs.202404900

**Published:** 2024-08-19

**Authors:** Yuting Fu, Le Chen, Yuao Guo, Yuqing Shi, Yanjun Liu, Yuqiang Zeng, Yuanjing Lin, Dan Luo

**Affiliations:** ^1^ Department of Electrical & Electronic Engineering Southern University of Science and Technology Xueyuan Road 1088, Nanshan District Shenzhen 518055 China; ^2^ School of Microelectronics Southern University of Science and Technology Shenzhen 518055 China; ^3^ State Key Laboratory of Optical Fiber and Cable Manufacture Technology Southern University of Science and Technology Shenzhen 518055 China; ^4^ Guangdong Provisional Key Laboratory of Functional Oxide Materials and Devices Southern University of Science and Technology Shenzhen 518055 China

**Keywords:** hexagonal boron nitride nanoplates, micro‐pyramid textured photonic films, radiative cooling, scattering fillers, thermal conductivity

## Abstract

Sub‐ambient cooling technologies relying on passive radiation have garnered escalating research attention owing to the challenges posed by global warming and substantial energy consumption inherent in active cooling systems. However, achieving highly efficient radiative cooling devices capable of effective heat dissipation remains a challenge. Herein, by synergic optimization of the micro‐pyramid surface structures and 2D hexagonal boron nitride nanoplates (*h*‐BNNs) scattering fillers, pyramid textured photonic films with remarkable solar reflectivity of 98.5% and a mid‐infrared (MIR) emittance of 97.2% are presented. The *h*‐BNNs scattering filler with high thermal conductivity contributed to the enhanced through‐plane thermal conductivity up to 0.496 W m^−1^ K^−1^ and the in‐plane thermal conductivity of 3.175 W m^−1^ K^−1^. The photonic films exhibit an optimized effective radiative cooling power of 201.2 W m^−2^ at 40 °C under a solar irradiance of 900 W m^−2^ and a daily sub‐ambient cooling effect up to 11 °C. Even with simultaneous internal heat generation by a 10 W ceramic heater and external solar irradiance of 500 W m^−2^, a sub‐ambient cooling of 5 °C can be realized. The synergic matching strategy of high thermal conductivity scattering fillers and microstructured photonic surfaces holds promise for scalable sub‐ambient radiative cooling technologies.

## Graphical abstract

In this work, pyramid‐textured photonic films with remarkable solar reflectivity and a mid‐infrared emittance are presented. The films can achieve sub‐ambient cooling performance up to 5 °C at internal heat generation and external solar irradiance. The synergic strategy of high thermal conductivity fillers and microstructured photonic ssurfacespromises to advance sub‐ambient radiative cooling technologies for efficient building‐scale applications.

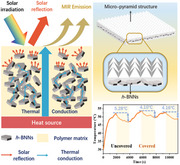

## 1 Introduction

The increasing heat generation and carbon emissions worldwide pose an urgent demand for effective cooling technologies. Currently, the predominant cooling systems are based on traditional active strategies that require continuous supplies of electricity and coolants.^[^ ^1^ ^]^ It is estimated that ≈10% of global electricity is consumed by building cooling, meanwhile generating a huge amount of hazardous gas emissions and carbon emission.^[^ ^2^ ^]^ Therefore, the development of eco‐friendly and passive cooling approaches without external power supply is of great significance. Radiative cooling emerges as one of the promising passive cooling technologies for various applications, including vehicles and buildings thermal regulation. It reflects heat from solar irradiation within the wavelength of 0.3–2.5 µm and dissipates the internal thermal energy to the cooler outer space via the atmospheric window within the wavelength of 8–13 µm.^[^ ^3^ ^]^ With rational photonic properties engineering to achieve high solar reflectance and MIR emittance simultaneously, reducing the temperature in the specific space to lower than ambient, which is known as sub‐ambient cooling, can be realized.^[^ ^4^ ^]^ However, for those practical scenarios such as communication base stations and data centers with tremendous heat generation, it remains a critical challenge to achieve sub‐ambient cooling in such a passive manner.^[^ ^5^ ^]^

To construct radiative cooling devices capable of highly efficient heat dissipation, photonic films with high reflectance/emittance factors and thermal conductivity are expected. Hybrid photonic films that consist of polymer matrix and high MIR emittance and scattering fillers, such as silicon dioxide, alumninium oxide, and titanium dioxide, have been reported in previous research.^[^ ^6^ ^]^ These fillers with a high‐refractive‐index normally have a band gap higher than 4.13 eV (i.e., the maximal photon energy in the solar spectrum) for minimal solar absorption. Nevertheless, too large a bandgap might also weaken the scattering and reflection due to the decreased refractive index. Among various candidates, boron nitride possesses a preferred bandgap of 5.96 eV, and a relatively large refractive index of 2.1–2.3 and 1.4–1.6 for the in‐ and cross‐plane orientation (Figure S1, Supporting Information). However, most of the photonic films have large thicknesses so as to realize sufficient scattering. Besides, the molecular structures of the polymer matrix normally result in low thermal conductivity. The unavoidable internal heat accumulation could suppress the radiative cooling effect.^[^ ^7^ ^]^ Therefore, it is necessary to form thermal conductive networks in the radiative photonic films.

**Figure 1:**
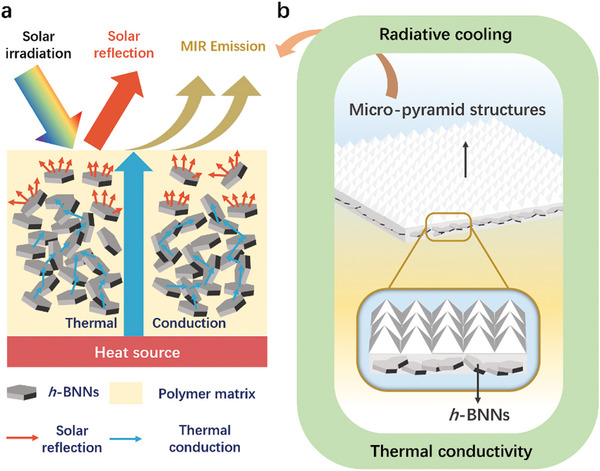
Schematic illustrating of the PTPFs with high‐refractive index fillers. a) PTPFs with high solar reflectivity, MIR emissivity, and thermal conductivity. b) PTPFs architecture with micro‐pyramid surface structure and *h*‐BNNs fillers.

In this work, highly efficient photonic films with pyramid array textures and high‐refractive two‐dimentional (2D) *h*‐BNNs fillers have been developed. Through synergic optimization of the surface photonic properties based on micro‐pyramid structures and high‐refractive index 2D *h*‐BNNs fillers, simultaneous enhancement of photonic selectivity and heat dissipation were achieved. The micro‐pyramid surface structures and *h*‐BNNs scattering fillers contribute to the effective sunlight backscattering at the surface region. Meanwhile, the *h*‐BNNs fillers enable thermal conduction through the polymer matrix network. With a systematic study of the rational matching between the structural factors, the radiative cooling films with desirable solar reflection, MIR emission, and thermal conduction are presented (**Figure** **1**a). The as‐developed pyramid textured photonic films (PTPFs) exhibit extremely high solar reflectivity of 98.5% and high MIR emittance of 97.2%. The through‐plane and the in‐plane thermal conductivity are up to 0.496 and 3.175 W m^−1^ K^−1^ respectively, which display significant enhancements compared to most of the reported polymer films. The PTPFs deliver an average effective radiative cooling power of up to 201.2 W m^−2^ at 40 °C under a solar irradiance of 900 W m^−1^. In a whole‐day demonstration with temperature variation from 22 to 43 °C, a sub‐ambient cooling effect of 8–11 °C was recorded. As a proof‐of‐concept, the PTPFs were applied on a building model with inner heat generation, demonstrating effective cooling with an inner temperature decrease of up to 5 °C under solar irradiance of 500 W m^−2^. The proposed strategy to realize highly efficient radiative photonic films by synergistic matching of thermal conductive 2D fillers and micro‐structured surfaces demonstrates a convenient route toward rational design of effective sub‐ambient cooling devices.

## 2 Results and Discussion

### 2.1 Preparation and Characterization of the Photonic Films

**Figure 2:**
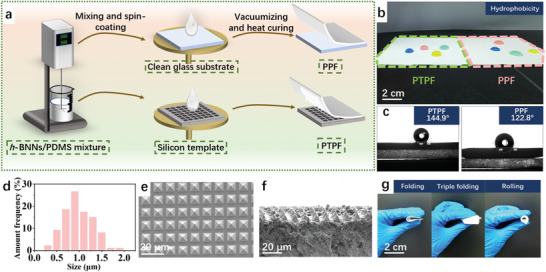
Fabrication and structural characterization of photonic films. a) The fabrication process of PTPF and PPF. b) The photographs of fabricated PTPFs and PPFs with water droplets on top. c) The contact angles of the PTPFs and PPFs. d) The size distribution of *h*‐BNNs in PTPFs. e,f) SEM images of the PTPFs depicting the well‐arranged micro‐pyramid arrays on the surface. g) Photographs of PTPF being folded, triple‐folded, and rolled.

The radiative photonic films were fabricated via a facile and scalable thermal curing method (**Figure** **2**a). The precursor solution consists of polydimethylsiloxane (PDMS) and *h*‐BNNs was first mechanically mixed into a homogenous state before being spin‐coated onto substrates. PDMS was selected due to its high solar transmittance and high infrared emittance (Figure S2, Supporting Information).^[^ ^8^ ^]^ Free‐standing photonic films can be peeled off after curing. The film thickness was optimized at ≈1 mm, controlled by the spin coating speed and duration. To evaluate the effectiveness of photonic engineering with textured surfaces, inverted micro‐pyramid silicon wafers were fabricated for micro‐structures soft transfer onto the PDMS. The micro‐pyramid structure was designed with a width of 8 µm and height of 5.7 µm, as optimized in prior studies.^[^ ^9^ ^]^ The planar photonic films (PPFs) were prepared based on cleaned glass substrates for comparison. Due to the strong broadband reflectance in the range of solar irradiation, the photonic films appear whitish (Figure 2a). It is also found that the as‐fabricated large‐scale PTPFs show a slightly increased hydrophobicity compared to the PPFs (Figure 2b,c). The uniformity of the as‐fabricated photonic films is also characterized. The optimized PTPFs contain *h*‐BNNs in an average size of 1 µm (Figure 2d), with a concentration of 60%. The uniform distribution of *h*‐BNNs can be confirmed by energy‐dispersive X‐ray (EDX) spectroscopy (Figure S3, Supporting Information). As shown in the SEM images in Figure 2e,f, the micro‐pyramid arrays can be well transferred. The PTPFs with such high concentrations of *h*‐BNNs maintain desirable flexibility under folding and rolling (Figure 2g), possibly attributed to the enhanced stress‐releasing behavior with the micro‐structures.^[^ ^10^ ^]^ It is specifically desirable for radiative cooling devices with potential thermal expansion mismatch in the hybrid compositions. Such a scalable strategy can also be applied for industrial fabrication, such as the roll‐to‐roll technique, to fulfill the requirement for smart curtains and building scale applications with promising self‐cleaning capability.

### 2.2 Optimization of the Radiative Cooling Films

**Figure 3:**
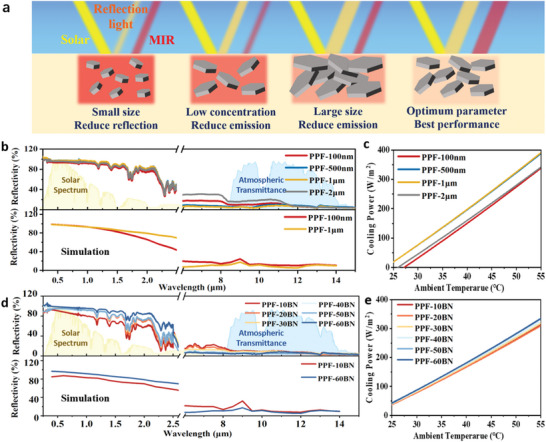
Optical properties and simulation modulated by the sizes and concentrations of *h*‐BNNs fillers. a) The schematic diagram of the solar reflection and the MIR absorption variation with different *h*‐BNNs fillers. The solar reflectance spectra, MIR absorption spectra, and corresponding simulation results varied with b) the size and d) the concentrations of *h*‐BNNs. The radiative cooling power of PPFs modulated by c) the size and e) the concentrations of *h*‐BNNs.

To optimize the radiative cooling performance, systematic experiments were first performed to investigate the impact of filler size and concentration on the reflectivity of planer photonic films (**Figure** **3**a). The h‐BNNs used in the experiment were all purchased in uniform sizes. the SEM images of different sizes and particle size distribution bar charts in Figure S4 (Supporting Information) illustrate the uniformity of h‐BNNs size in the PPFs. To evaluate the impact of filler sizes, *h*‐BNNs in the range of 100 nm to 2 µm are utilized in the concentration of 60%. Under the solar spectrum, *h*‐BNNs in comparable size (≈1 µm) result in high reflectance due to the strong Mie scattering, while the small fillers (e.g., 100 nm) fail to effectively scatter the sunlight of wavelengths larger than 1 µm (Figure 3b). In the atmospheric window, the 1 µm filler size leads to the highest emissivity for a weak scattering and strong absorption. In contrast, the photonic films based on 2 µm filler size have the lowest emissivity due to the relatively strong scattering even in the MIR range. The theoretical reflectance and absorptance were calculated by solving the Radiative Transfer Equation, which agrees well with the measurement results.^[^ ^11^ ^]^ As verified by the radiative cooling power tests for PPFs with different filler sizes and at varied ambient temperatures (Figure 3c), the optimized filler size is determined at 1 µm.

Moreover, the filler concentration effect on the radiative cooling performance was evaluated. The optimal *h*‐BNNs in the size of 1 µm were adopted to prepare photonic films with filler concentrations ranging from 10% to 60%. The filler concentration higher than 60% could result in a viscous solution and nonuniform films. It is observed that as the filler concentration increases, the stronger scattering and reflection in the solar spectrum lead to enhanced reflectivity (Figure 3d). In the MIR range, the long‐wavelength photons can be absorbed by the PDMS, and thus the stronger scattering with the increased filler concentration results in stronger absorption and higher absorptivity/emissivity. Similarly, the calculated reflectance and absorptance for different filler concentrations follow a good agreement with the experimental data. Therefore, it can be concluded that the higher filler concentrations lead to enhanced radiative cooling power (Figure 3e). The cooling power of the as‐prepared PPFs reaches 198.1 W m^−2^ at 40 °C ambient temperature with a solar irradiance of 900 W m^−2^.

**Figure 4:**
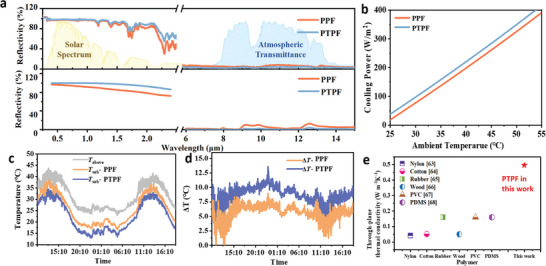
Optical properties and radiative cooling performance modulated by the micro‐pyramid arrayed surface structure. a) The solar reflectance spectra and the MIR absorption spectra of PPFs and PTPFs, and the corresponding simulated results. b) The radiative cooling power of PPFs and PTPFs. c,d) The temporal temperature data of *T*_above_ and *T*_sub_ during outdoor radiative cooling measurement with PPFs and PTPFs respectively. e) The through‐plane thermal conductivity of the PTPFs in this work compared with common polymers.

In addition to the filler size and concentration optimization, the micro‐pyramid structures on the photonic films were applied to further improve the radiative cooling performance.^[^ ^4b^ ^]^ **Figure** **4**a shows a comparison of reflectance spectra between PPFs and PTPFs. In the solar spectrum, the pyramid boundaries enhance Mie scattering and internal reflection as the pyramid size with a width of 8 µm and height of 5.7 µm is comparable to the sunlight wavelength. This contributes to the increase of total reflection as both the PDMS and fillers cannot effectively absorb the light at such short wavelengths. In contrast, PDMS can effectively absorb long‐wavelength light (e.g., in the MIR range), resulting in an increase of absorption/emission in this regime. By co‐optimizing the filler size, concentration, and the surface structures, the solar reflectivity and the MIR absorptivity of the PTPFs reaches 98.5% and 97.2%, respectively. As shown in Figure 4b, the cooling power is 220.1 W m^−2^ at 40 °C under a solar irradiance of 900 W m^−2^, which is 22 W m^−2^ higher than that of PPFs.

To evaluate the cooling performance of the optimal PTPFs for practical applications, an outdoor radiative cooling measurement system was built (Figure S5, Supporting Information). The ambient temperature and film temperature were measured using multiple thermocouples. Figure 4c displays the temporal evolution of temperature for the cases with PPFs (*T_PPFs_*) and PTPFs (*T_PTPFs_*) under a total solar irradiation of *I*_solar_ ≈ 800 W m^−2^ and a relative humidity of 60%. The comparison of the temperature drop (*T_a_*−*T_PTPFs_* vs *T_a_*−*T_PPFs_*) demonstrated the improved all‐day radiative cooling performance is shown in Figure 4d. With the PTPFs, the improved cooling effectiveness of 4 °C at noon and 5 °C during the night compared to the PPFs were recorded.

Apart from the desired optical properties, the significantly enhanced thermal conductivity with the *h*‐BNNs fillers is critical for efficient heat dissipation. The thermal conductivity of most polymer matrices (e.g., nylon, cotton, PVDF, PVC, PDMS, wood, rubber) is in the range of 0.1–0.2 W m^−1^ K^−1^. The through‐plane thermal conductivity and the in‐plane thermal conductivity of PPFs with *h*‐BNNs fillers concentration increasing are presented in Figure S6 (Supporting Information). Obviously, both the through‐plane thermal conductivity and the in‐plane thermal conductivity of PPFs increase rapidly with the thermally conductive fillers concentrations. It can be well understood using the effective medium theory (e.g., Bruggemann model).^[^ ^12^ ^]^ With a filler concentration of 60%, the thermal conductivity reaches 0.5 W m^−1^ K^−1^ for the through‐plane direction (Figure 4e), which displays a 2.5–5 times improvement. The high through‐plane thermal conductivity would benefit the heat dissipation in practical scenarios with internal heat generation sources.

### 2.3 Demonstration of PTPFs for Effective Cooling

**Figure 5:**
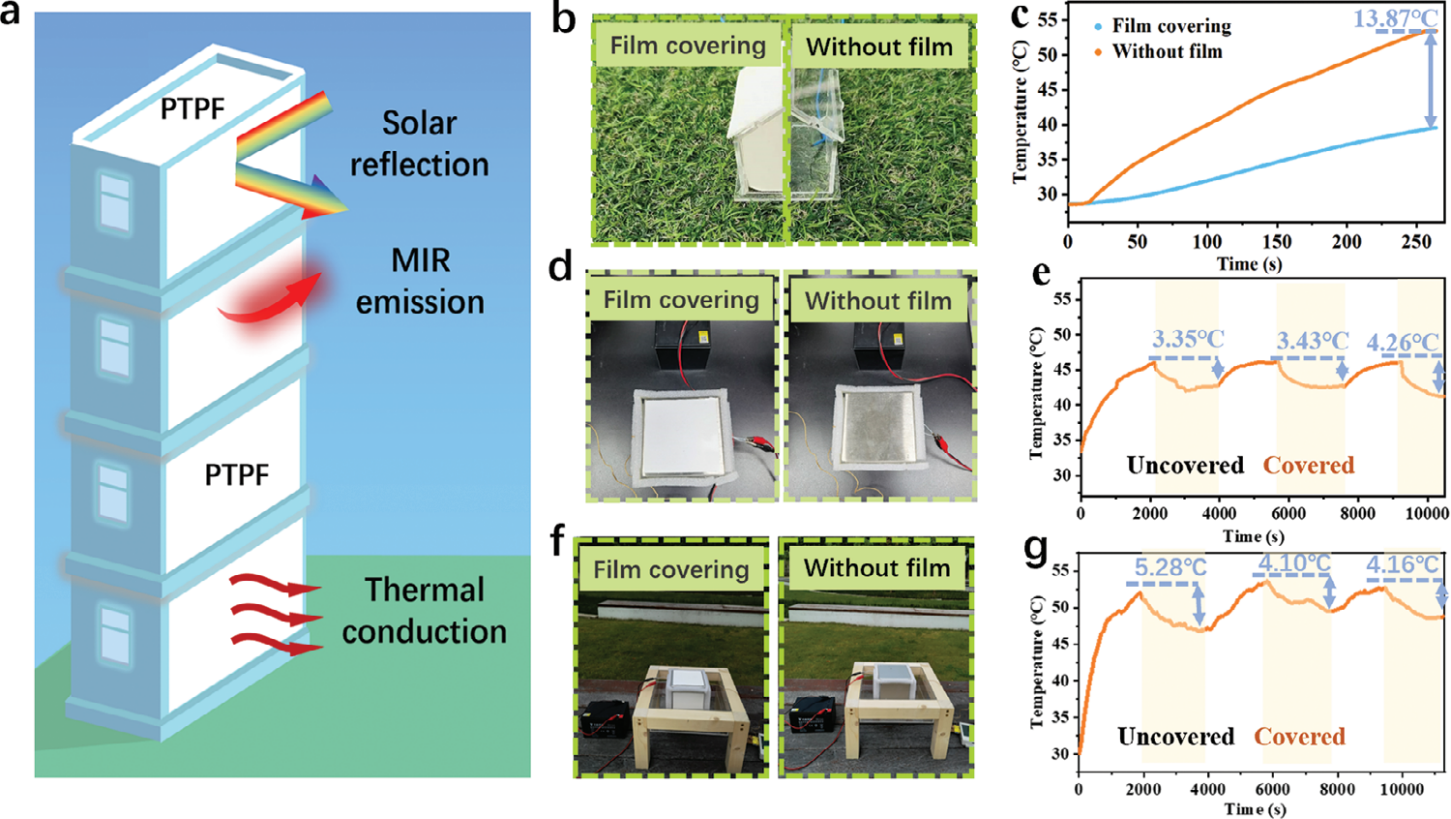
Radiation cooling demonstrations of the PTPFs. a) Schematic diagram of the radiative cooling applications of PTPFs on buildings. b) The pictures showing the outdoor measurement under solar irradiation. c) The temperature variation in the mini house model with and without PTPFs covering. d) The pictures showing the indoor measurement for thermal conduction. e) The sub‐ambient equilibrium temperature variation measured indoor when PTPFs were applied and taken off. f) The pictures showing the outdoor measurement for radiative cooling under solar irradiation and with internal heat sources. g) The sub‐ambient equilibrium temperature variation measured under the solar irradiation when PTPFs were applied and taken off.

The proposed PTPFs with desirable fabrication scalability, flexibility, and remarkably enhanced radiative cooling performances show the promise for effective building‐scale cooling (**Figure** **5**a). As a proof‐of‐concept, sub‐ambient cooling demonstrations with and without solar irradiation and internal heat generation were performed respectively.

In the case without internal heat generation, the thermal energy input mainly comes from solar irradiation. To maximize the temperature rise due to this effect, a transparent acrylic mini house model (length × width × height: 4 × 4 × 4.5 cm) was built to ensure maximal sunlight transmission through the roof and walls (Figure 5b). The internal temperature variation of the mini house model covered with and without PTPFs is shown in Figure 5c. Under 250 s of exposure to the natural solar irradiation of *I*_solar_ = 549 W m^−2^, the internal temperature with PTPFs reaches 13.87 °C lower than the bare ones.

In most of the other scenarios, there are heat generation sources inside the buildings, such as with human activities and machine operation. To realize efficient heat conduction through the wall and then effective dissipation to the external environment, the utilization of thermally conductive materials is of significance. To validate the heat dissipation capability of the PTPFs, a cubic stainless‐steel box (side width: 10 mm) with a constant heat source powered using a 12 V battery in the center was adopted for demonstration (Figure 5d). Stainless steel was selected for its relatively high thermal conductivity (≈15 W m^−1^ K^−1^) to eliminate the interference by low thermal resistance blocking. Indoor tests without solar irradiation were first performed. Compared to the bare box, the temperature drop with our PTPFs is in the range of 3–4 °C. Afterward, outdoor experiments were then conducted under the solar irradiation of *I*_solar_ ≈500 W m^−2^ and a relative humidity of 29%. Figure 5g shows a slightly increased temperature drop of 4–5 °C, which indicates the effectively enhanced radiative cooling efficiency of the PTPFs by combining the superiorities of high thermal conductivity and optimal optical properties. These demonstrations highlight that the as‐fabricated PTPFs have broad applications for sub‐ambient cooling regardless of the variation of solar irradiation, internal heat generation, and intrinsic thermal conductivity of the applied subjects.

## 3 Conclusion

In summary, we proposed a facile and scalable strategy to fabricate high‐performance photonic films with largely enhanced thermal conductivity via the synergic optimization of thermal conductive *h*‐BNNs scattering fillers and micro‐pyramid photonic surface structures. Compared to radiative cooling photonic films of recent years, a remarkably high solar reflectivity of 98.5% and MIR emittance of 97.2% were achieved, which are among the best (Table S1, Supporting Information). The PTPFs presented an average effective radiative cooling power of 201.2 W m^−2^ at 40 °C under a solar irradiance of 900 W m^−2^ and a sub‐ambient cooling effect of 8–11 °C in a day without internal heat generation. Moreover, the through‐plane thermal conductivity and the in‐plane thermal conductivity of the PTPFs were up to 0.496 and 3.175 W m^−1^ K^−1^ respectively, which deliver significant enhancement compared to most of the polymer films. Thus, under the scenarios with internal thermal energy generation, the PTPFs demonstrated a sub‐ambient cooling performance of up to 5 °C under a solar irradiance of 500 W m^−2^. The rational optimization and in‐depth theoretical study to realize photonic films with high thermal conductivity and micro‐textured surfaces provide an effective strategy to achieve sub‐ambient radiative cooling with high efficiency.

## 4 Experimental Section

### Materials

All the chemicals were reagent grade and used as received. PDMS precursor was Sylgard 184 (Dow Corning Co.). *h*‐BNNs were purchased from the Shanghai Chaowei Nanotechnology Co., Ltd., China. The K‐type thermocouples and the data logger were provided by HOBO UX120‐014M, Onset Computer Corp., USA.

### Fabrication of the Inverted Micro‐Pyramid Silicon Wafer

The inverted micro‐pyramid silicon wafer was prepared by wet etching. In the first step, the silicon oxide wafer was cleaned by ethyl alcohol, acetone, and deionized water, and heated up to 125 °C for drying subsequently. Then, the photoresist was evenly applied to the silicon oxide wafer and baked at 115 °C for 1 min. The lithographic mask was designed as square pores arrays, where the size of square pores was fixed at 8 × 8 µm. After the lithography process, reactive ion etching was conducted on the silicon wafer to eliminate the exposed silicon nitride layer and release the underlying silicon substrate. The silicon wafer underwent wet‐etching to undergo anisotropic treatment. The formulation of the silicon corrosion solution consists of a blend of potassium hydroxide, isopropyl alcohol, and water in a ratio of 1:2:2. After the etching solution reached the bottom tip of the silicon, the chemical reaction ceased. Subsequently, the etched silicon wafer underwent a cleaning process to eliminate any residual photoresist and silicon nitride from its surface. The template was finally coated with a layer of fluorocarbon film to facilitate the easy removal of the photonic films from the silicon chip.

### Fabrication of the Photonic Films

The precursor solution consisted of PDMS and varied‐sized/concentrated *h*‐BNNs were obtained via intense mechanical agitation and spin‐coated onto well‐cleaned substrates. The film thickness can be controlled by varying the speed and time of spin‐coating. A well‐cleaned glass substrate was used to prepare planar photonic films, and an inverted micro‐pyramid silicon wafer was designed as the template to prepare PTPFs. The silicon wafer was prepared via traditional photolithography, dry etching, and wet etching. The precursors coated on the templates were vacuumed to remove air bubbles for over 60 min at room temperature, followed by 90 °C heating for 2 h. Afterward, the samples were cooled down to room temperature for film solidification. Free‐standing photonic films can be achieved after peeling off from the substrates.

The average sizes of *h*‐BNNs were 100 nm, 500 nm, 1 µm, and 2 µm, and the corresponding photonic films were labeled as film‐100 nm, 500 nm, 1 µm, and 2 µm. The ratio of *h*‐BNNs to the PDMS and curing agent was defined as x:1:0.1, in which x can be 10%, 20%, 30%, 40%, 50%, and 60% mass ratio. The photonic films were named as film‐10BN, 20BN, 30BN, 40BN, 50BN, and 60BN. For the planar photonic films, *h*‐BNNs in the size of 1 µm were adopted, with a mass ratio of 60 wt% in the precursor solution.

### Characterizations

The morphology and structure of the PTPFs were characterized by field‐emission scanning electron microscopy (Zeiss Sigma 500). Reflectance measurements of the PPFs and PTPFs in the wavelength range of 0.3–2.5 µm were recorded using a Lambda 750s UV–vis–NIR spectrophotometer equipped with an integrating sphere (PerkinElmer Inc.). The transmittance and reflectance in the wavelength range of 2.5–14 µm were conducted on a Fourier transform infrared spectrometer (Vertex 70v, Bruker, with a gold integrating sphere (Model 4P‐GPS‐020‐SL, Labsphere).

The through‐plane thermal conductivity and the in‐plane thermal conductivity were both obtained via:

(1) $$\lambda \;=C_{p}\;\rho \alpha$$

where λ is the thermal conductivity, *C*_P_ is the specific heat capacity, ρ is the density, and α is the thermal diffusivity. The specific heat capacity of the sample was measured by DSC214 provided by NETZSCH Scientific Instruments Trading (Shanghai) Ltd. The density of the sample was provided by D4 densimeter (Mettler Toledo Instruments (Shanghai) Co., Ltd.). The thermal diffusivity of samples was obtained by LFA467 laser thermal conductivity measuring instrument (NETZSCH Scientific Instruments Trading (Shanghai) Ltd.). The contact angle of pure PDMS film, PPFs, and PTPFs was measured by the Standard optical contact goniometer (SL200B, USA KINO Industry Co., Ltd.).

### Outdoor Measurement for Radiative Cooling Without an Internal Heat Source

The outdoor measurement for radiative cooling of PPFs and PTPFs without an internal heat source was accomplished via equipment constituted of an aluminum foil‐coated wooden frame chamber with a polystyrene foam block. The polystyrene foam block was 15 cm high and 20 × 20 cm wide, placed in a wooden frame with external dimensions of 20 × 30 × 30 cm. The polystyrene foam block was embedded in the wooden frame chamber without any gaps to reduce convective heat loss and improve thermal isolation. The wooden frame was covered by an aluminum foil to prevent heating from sunlight. The film to be measured was placed in the 10 × 10 cm square groove in the center of the foam block with contacting edges as small as possible to minimize the heat exchange.

In the demonstration with a transparent acrylic mini house model, the model was designed in 4.5 cm high and 4 × 4 cm^2^ wide. The transparent acrylic mini house model was symmetrically divided into two sections, one of which was covered by PTPFs with the same specifications for the roof and the walls, while the other part was not uncovered. In order to ensure the same light intensity in both sections, the transparent acrylic mini‐house model was exposed to the sunlight along the center axis.

To measure the real‐time temperature variation of the sub‐ambient and above‐ambient of the measured film, two K‐type thermocouples connected to temperature recorders were placed in the equipment and recorded the data utilizing a data logger (HOBO UX120‐014M, Onset Computer Corp.). The measured film was completely exposed to the ambient air without any shelter when tested to avoid forming a microclimate different from the ambient air. The comparison between PPFs and PTPFs was conducted in Shenzhen, China (22°32′44′′N, 114°3′10′′E) on April 12th, 2023 under a total solar irradiation of *I*_solar_ ≈ 800 W m^−2^, a temperature of 27.2 °C and a relative humidity of 60%. The demonstration on a mini house model to compare the cooling performance with and without PTPFs was under the solar irradiation of *I*_solar_ = 549 W m^−2^ and a temperature of 32.2 °C in Shenzhen, China on July 2nd, 2023.

### Thermal Conduction Measurement

A rectangular stainless‐steel box with a side length of 10 cm was utilized to measure the thermal conduction of the PTPFs. Three connect planes of the box were covered with 2 ‐mm‐thick polystyrene foam while the others were covered by PTPFs with the same specifications. The gaps between each two PTPFs were filled with polystyrene foam on the edges of the box to reduce heat loss. A constant heat source was provided inside the stainless‐steel box by a ceramic heater (10 W) powered by a 12 V battery. A K‐type thermocouple detecting the heated sub‐ambient temperature was placed inside the box and recorded the data utilizing a data logger. Outdoor experiments were then conducted under the solar irradiation of *I*_solar_ ≈ 500 W m^−2^, a temperature of 30 °C and a relative humidity of 29% in Shenzhen, China on May 18th, 2023.

### Outdoor Measurement for Radiative Cooling with an Internal Heat Source

The outdoor measurement equipment for radiative cooling with an internal heat source was similar to the thermal conduction measurement equipment. Under solar irradiation in the open air, the stainless‐steel box was placed on a suspended acrylic tray attached to a wooden frame, ensuring that the equipment was not affected by other heat exchange. A constant heat source was provided inside the stainless‐steel box by a ceramic heater (10 W) powered by a 12 V battery. A K‐type thermocouple detecting the heated sub‐ambient temperature was placed inside the box and recorded the data utilizing a data logger.

### Calculation of the Average Radiative Cooling Power

The average cooling power *P*_cool_(*T*) of PPF/PTPF is related to the blackbody radiation, thermal exchange with the atmosphere, solar irradiation, and heat exchange channels through conduction and convection:

(2) $$P_{\mathrm{cool}}\left(T\right)=P_{\mathrm{rad}}\left(T\right)-P_{\mathrm{atm}}\left(T_{\mathrm{amb}}\right)-P_{\mathrm{sun}}-P_{\mathrm{cc}}\left(T,T_{\mathrm{amb}}\right)$$

where *P*_rad_(*T*) is the power radiated by PPF/PTPF, *P*_atm_(*T*_amb_) is the power of the incident atmospheric radiation by atmospheric heat exchange outside the AW region, *P*_sun_ is the absorbed incident power from the sun, *P*_cc_(*T, T*_amb_) is the conduction and convection power, 𝑇 is the temperature of the radiative cooling device, and 𝑇_amb_ is ambient temperature.

The radiation power *P*_rad_(*T*) is the power radiated by PPF/PTPF film:

(3) $$P_{\mathrm{rad}}\left(T\right)=A\int d\Omega \cos\theta \int_{0}^{\infty }d\lambda I_{\mathrm{BB}}\left(T,\lambda \right)\varepsilon \left(\lambda ,\theta \right)$$

where *A*, *λ*, *θ* and $\int d\Omega =2\pi \int_{0}^{\pi /2}\sin\theta d\theta$ are the surface area of the sample, the wavelength, polar angle, and angular integral over a hemisphere, respectively.

The blackbody‐specific intensity *I*_BB_(*T, λ*) at *T* is

(4) $$I_{\mathrm{BB}}\left(T,\lambda \right)=\frac{2hc^{2}}{\lambda^{5}}\;\frac{1}{e^{\frac{hc}{\left(\lambda k_{B}T\right)}}-1}$$

where *h* is Plank's constant, *k*_B_ is the Boltzmann constant, *c* is the speed of light in vacuum, *λ* is the wavelength, and *ε*(*λ*, *θ*) is the spectral and angular emissivity of PPF/PTPF.

The power of the incident atmospheric radiation *P*_atm_(*T*_amb_) by atmospheric heat exchange outside the AW region is:

(5) $$P_{\mathrm{atm}}\left(T_{\mathrm{amb}}\right)=\int \int_{0}^{\infty }I_{\mathrm{BB}}\left(T_{\mathrm{amb}},\lambda \right)\varepsilon \left(\lambda ,\theta \right)\varepsilon_{\mathrm{atm}}\left(\lambda ,\theta \right)d\lambda \cos\theta d\Omega$$

where ε_atm_ (λ,θ) =  1 − *t*(λ)^1/cos θ^ and *t*(*λ*) are the atmospheric emissivity and the atmospheric transmittance in the zenith direction, respectively.

The absorbed incident power from the sun *P*_sun_ can be expressed as:

(6) $$P_{\mathrm{sun}}=A\int_{0}^{\infty }d\lambda \varepsilon \left(\lambda ,\theta_{\mathrm{sun}}\right)I_{AM1.5}\left(\lambda \right)$$

where *I*_AM1.5_ is the solar illumination intensity.

According to Newton's law, the conduction and convection power *P*_cc_(*T, T*_amb_) is:

(7) $$P_{\mathrm{cc}}\left(T,T_{\mathrm{amb}}\right)=Ah_{\mathrm{cc}}\left(T_{\mathrm{amb}}-T\right)$$

where *h*_cc_ the coefficient of the nonradiative transfer mechanisms, including heat conduction and convection. In this work, *h*_cc_ is deeply influenced by the thermal conductivity of PPF/PTPF.

### FDTD Optical Simulation

For the planar photonic films, the reflectivity and emissivity were calculated by solving the radiative transfer equation with a Monte Carlo solver.^[^ ^13^ ^]^ The optical properties associated with the *c*‐axis were used in the simulation for the nanoplate geometry of *h*‐BNNs particles. For particles with certain shapes (e.g., sphere), the randomized orientation should be carefully considered as discussed previously.^[^ ^14^ ^]^ The efficiencies and coefficients (extinction, scattering, and absorption) for each concentration were calculated based on Mie theory.^[^ ^15^ ^]^ As for the films with micro‐pyramid structures, a finite element analysis was performed to evaluate its effect on the optical properties (COMSOL Multiphysics 5.5, Wave optics physics). Since highly periodic microstructures were fabricated in the work, a periodic unit cell was used in the simulation, assuming negligible effect of random structures (Figure S7, Supporting Information). The top, bottom, and other boundaries were set as port 1, 2, and periodic boundary conditions. The reflectance (*R*) and transmittance (*T*) for TM and TE incident waves were calculated and the average was taken as the effective results for unpolarized light. Thus, the emittance of the micro‐pyramid structures can be calculated as:

(8) E=1−R−T

## Conflict of Interest

The authors declare no conflict of interest.

## Author Contributions

Y.F. and L.C. contributed equally to this work. D.L., Y. Lin., Y.Z., Y.F., and Y.G. conceived the study. Y.F. performed the film fabrication, optimization, characterization, demonstration, and data analysis with assistance from L.C. Y.Z. performed the simulations. Y.F. and L.C. performed the schematic drawing and data plotting. D.L., Y. Lin., Y.Z., Y.F., L.C., Y.G., Y. S., and Y. Liu wrote and revised the manuscript. All authors provided feedback on the results and the paper.

## Supporting information

Supporting Information

## Data Availability

The data that support the findings of this study are available from the corresponding author upon reasonable request.
