# Correction to “Pyramid Textured Photonic Films with High‐Refractive Index Fillers for Efficient Radiative Cooling”

**DOI:** 10.1002/advs.202514484

**Published:** 2025-08-12

Yuting Fu^#^, Le Chen^#^, Yuao Guo, Yuqing Shi, Yanjun Liu, Yuqiang Zeng^*^, Yuanjing Lin^*^, Dan Luo^*^. Pyramid textured photonic films with high‐refractive index fillers for efficient radiative cooling. *Advanced Science*. 2024, 11(39): 2404900

https://doi.org/10.1002/advs.202404900

The affiliation listed for author Yuqing Shi was incomplete. It originally stated only “School of Microelectronics, Southern University of Science and Technology, Shenzhen 518055, China.” This should be: “School of Microelectronics, Southern University of Science and Technology, Shenzhen 518055, China, and Department of Applied Biology and Chemical Technology, The Hong Kong Polytechnic University, 999077, Hong Kong SAR, China.”

We apologize for this error

