# Supplementary material for: Pyramid Textured Photonic Films with High‐Refractive Index Fillers for Efficient Radiative Cooling
Source: Adv Sci (Weinh). 2024 Aug 19;11(39):2404900. doi: 10.1002/advs.202404900 (PMC11497053; doi:10.1002/advs.202404900)
Supplement: Supplementary file 1 — Supporting Information [file ADVS-11-2404900-s001.docx]

Supporting Information

Pyramid textured photonic films with high-refractive index fillers for efficient radiative cooling

*Yuting Fu^1,#^, Le Chen^2,#^,Yuao Guo^1^, Yuqing Shi^2^, Yanjun Liu^1^, Yuqiang Zeng^2,*^, Yuanjing Lin^2,*^, Dan Luo^1,3,4*^*

1 Department of Electrical & Electronic Engineering, Southern University of Science and Technology, Xueyuan Road 1088, Nanshan District, Shenzhen, 518055, China.

2 School of Microelectronics, Southern University of Science and Technology, Shenzhen 518055, China.

3 State Key Laboratory of Optical Fiber and Cable Manufacture Technology, Southern University of Science and Technology, Shenzhen, 518055, China.

4 Guangdong Provisional Key Laboratory of Functional Oxide Materials and Devices, Southern University of Science and Technology, Shenzhen, 518055, China.

*Corresponding author. Email: luod@sustech.edu.cn (D. L.); linyj2020@sustech.edu.cn (Y. L.); zengyq@sustech.edu.cn (Y. Z.)

**Figure S1.** The band gap and the refractive index at a wavelength of 500 nm of *h*-BNNs and some common scattering fillers^[1]^.

**Figure S2.** The polydimethylsiloxane (PDMS) has high-solar-transmittance (a) and high-infrared-emittance (b).


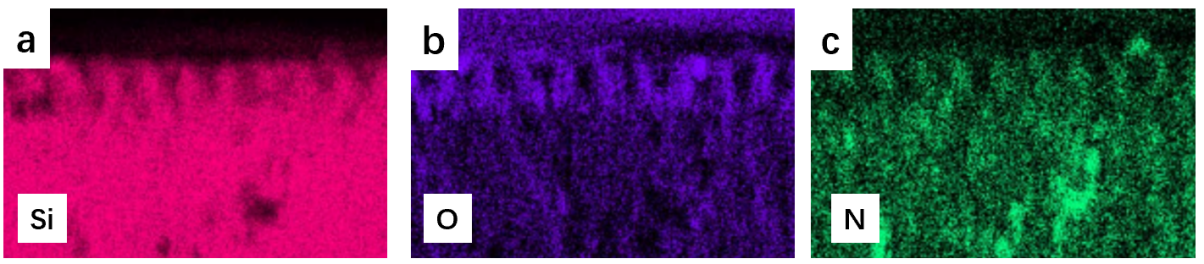


**Figure S3.** (a-c)The elemental mapping images measured by Energy-dispersive X-ray spectroscopy (EDX) of the BRCP film.


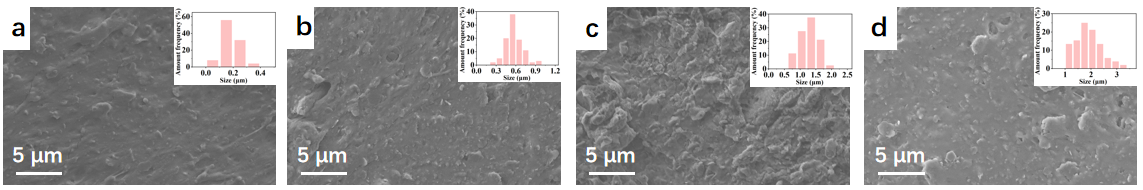


**Figure S4.** SEM images of the PPFs with h-BNNs size of 100 nm, 500 nm, 1 μm and 2 μm.


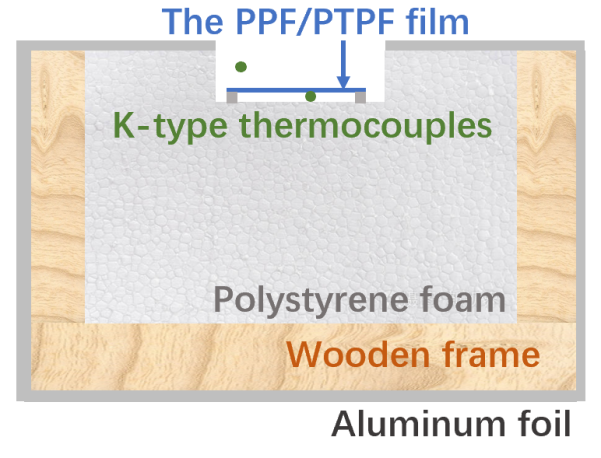


**Figure S5.** The outdoor radiative cooling measurement system constitutive of polystyrene, wood and Aluminum foil.

**Figure S6.** The
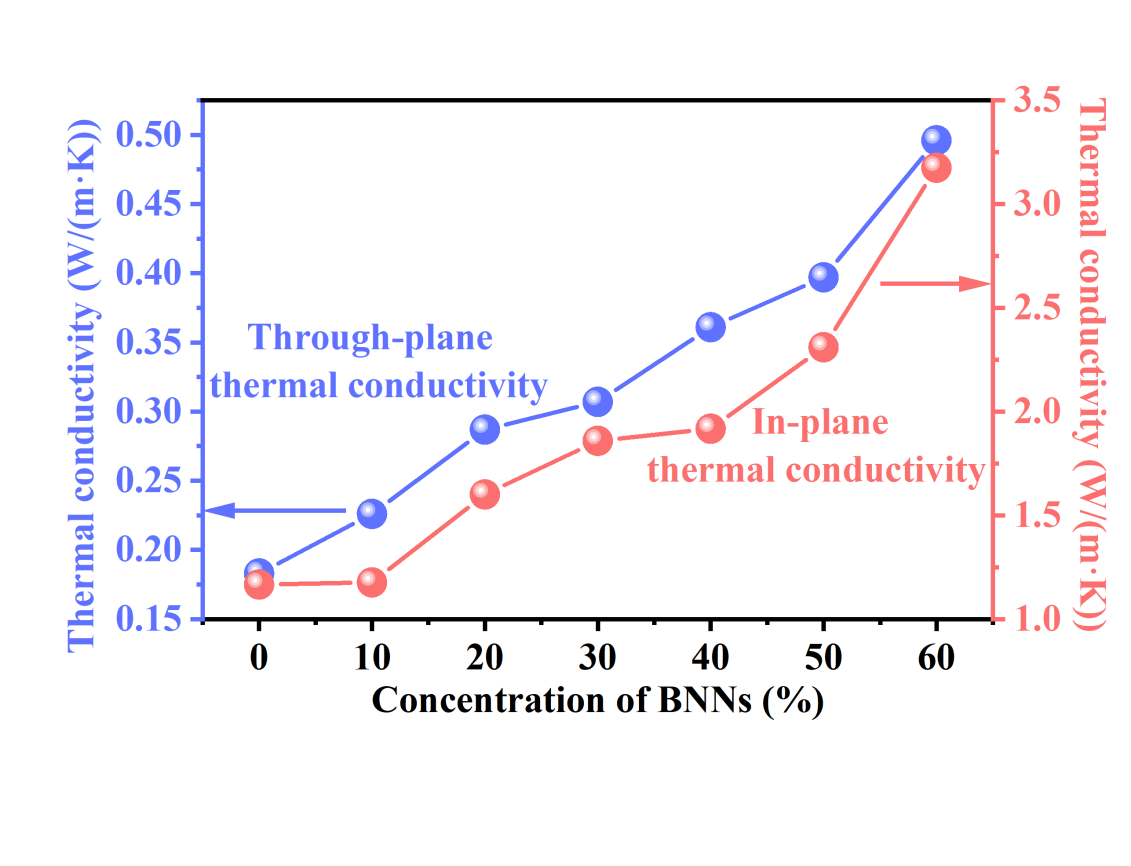
 through-plane thermal conductivity and the in-plane thermal conductivity of PPFs with *h*-BNNs concentration increasing.


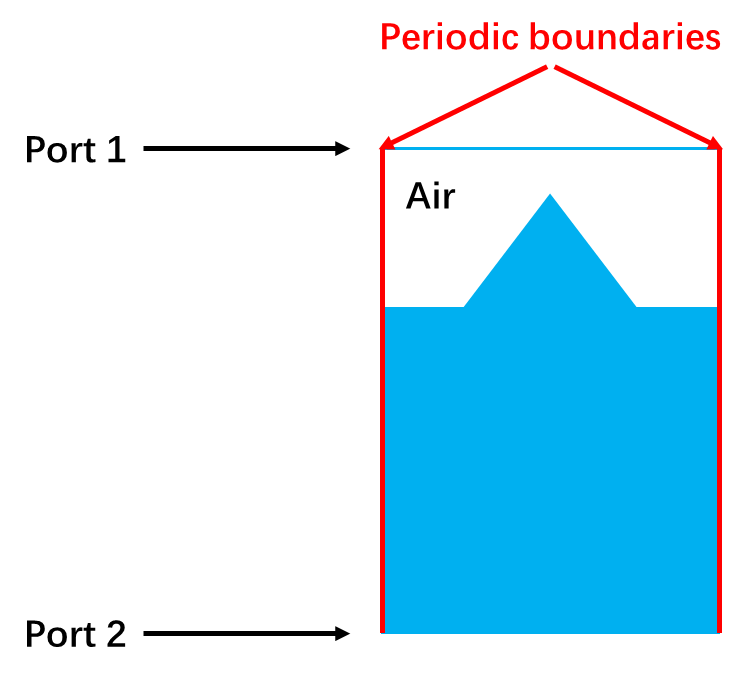


**Figure S7.** The unit cell and boundary conditions used in the calculation of reflectance and transmittance for the films with micro-pyramid structures.

| Material | Structure | Thermal conductivity  (W m^-1^ K^-1^) | Solar reflectivity | MIR |
| --- | --- | --- | --- | --- |
| Al_2_O_3_^[2]^ | Nano-processed silk | 0.502 | 95% | 90% |
| PVA-CaCl_2_^[3]^ | CA fibrous network | N/A | 95% | 94% |
| HIRC^[4]^ | Hierarchically architected metapaper | 0.12 | 99% | 90% |
| PVDF^[5]^ | Hierarchically hollow microfibers | 0.014 | 94% | 94% |
| K_2_Ti_6_O_13_^[6]^ | Nanocomposite PEO film | N/A | 94% | 91% |
| SiO_2_^[7]^ | Triple-layer structure | 0.035 | 96.4% | 94.6% |
| BN nanosheets ^[8]^ | Anisotropic cooling aerogel | 0.0169 | 97% | 90% |
| PET^[9]^ | Hierarchically porous film | 0.724 | 90.1% | 94.3% |
| PVDF-C_3_F_6_ ^[10]^ | Particles-based poly sponge | 0.048 | 94.5% | 95.6% |
| Al_2_O_3_^[11]^ | Hierarchical metafabric | 0.386 | 92% | 97% |
| PLA^[12]^ | Nanofabric | N/A | 99% | 91.2% |
| PMMA^[13]^ | 3D interconnected ordered porous polymer | 0.044 | 94% | 95% |
| **This work** | **Micro-pyramid structure** | **0.496** | **98.5%** | **97.2%** |

**Table S1.** Comparison of different radiative cooling photonic films

**References**

[1] a)Z. Tong, J. Peoples, X. Li, X. Yang, H. Bao, X. Ruan, *Mater. Today Phys.* **2022**, 24, 100658; b)N. M. Ravindra, P. Ganapathy, J. Choi, *Infrared Phys. Techn.* **2007**, 50, 21; c)J. Mandal, Y. Yang, N. Yu, A. P. Raman, *Joule* **2020**, 4, 1350; d)H. Bao, C. Yan, B. Wang, X. Fang, C. Y. Zhao, X. Ruan, *Sol. Energy Mater. Sol. C* **2017**, 168, 78; e)G. Cassabois, P. Valvin, B. Gil, *Nat. Photonics* **2016**, 10, 262; f)Y. Rah, Y. Jin, S. Kim, K. Yu, *Opt. Lett.* **2019**, 44, 3797.

[2] B. Zhu, W. Li, Q. Zhang, D. Li, X. Liu, Y. Wang, N. Xu, Z. Wu, J. Li, X. Li, P. B. Catrysse, W. Xu, S. Fan, J. Zhu, *Nat. Nanotechnol.* **2021**, 16, 1342.

[3] J. Li, X. Wang, D. Liang, N. Xu, B. Zhu, W. Li, P. Yao, Y. Jiang, X. Min, Z. Huang, S. Zhu, S. Fan, J. Zhu, *Sci. Adv.* **2022**, 8, eabq0411.

[4] Y. Tian, X. Liu, Z. Wang, J. Li, Y. Mu, S. Zhou, F. Chen, M. L. Minus, G. Xiao, Y. Zheng, *Nano Energy* **2022**, 96, 107085.

[5] H. Zhong, Y. Li, P. Zhang, S. Gao, B. Liu, Y. Wang, T. Meng, Y. Zhou, H. Hou, C. Xue, Y. Zhao, Z. Wang, *ACS Nano* **2021**, 15, 10076.

[6] P. Yao, Z. Chen, T. Liu, X. Liao, Z. Yang, J. Li, Y. Jiang, N. Xu, W. Li, B. Zhu, J. Zhu, *Adv. Mater.* **2022**, 34, 2208236.

[7] C. Lin, Y. Li, C. Chi, Y. S. Kwon, J. Huang, Z. Wu, J. Zheng, G. Liu, C. Y. Tso, C. Y. H. Chao, B. Huang, *Adv. Mater.* **2022**, 34, 2109350.

[8] K.-Y. Chan, X. Shen, J. Yang, K.-T. Lin, H. Venkatesan, E. Kim, H. Zhang, J.-H. Lee, J. Yu, J. Yang, J.-K. Kim, *Nat. Commun.* **2022**, 13, 5553.

[9] Q. Tian, X. Tu, L. Yang, H. Liu, Y. Zhou, Y. Xing, Z. Chen, S. Fan, J. Evans, S. He, *Small* **2022**, 18, 2205091.

[10] M. Qin, H. Han, F. Xiong, Z. Shen, Y. Jin, S. Han, A. Usman, J. Zhou, R. Zou, *Adv. Funct. Mater.* **2023**, 33, 2304073.

[11] C. Fan, Y. Zhang, Z. Long, A. Mensah, Q. Wang, P. Lv, Q. Wei, *Adv. Funct. Mater.* **2023**, 33, 2300794.

[12] S. Feng, L. Yao, X. Chen, C. Liu, X. Bu, Y. Huang, M. He, Y. Zhou, *J. Colloid Interface Sci.* **2023**, 648, 117.

[13] Y. Zhang, T. Wang, X. Mei, M. Chen, L. Wu, *ACS Photonics* **2023**, 10, 3124.
